# Genetic Transformation of *Artemisia carvifolia* Buch with *rol *Genes Enhances Artemisinin Accumulation

**DOI:** 10.1371/journal.pone.0140266

**Published:** 2015-10-07

**Authors:** Erum Dilshad, Rosa Maria Cusido, Karla Ramirez Estrada, Mercedes Bonfill, Bushra Mirza

**Affiliations:** 1 Department of Biochemistry, Faculty of Biological sciences, Quaid-i-Azam University, Islamabad, Pakistan; 2 Laboratorio de Fisiología Vegetal, Facultad de Farmacia, Universidad de Barcelona, Spain; Nanjing Agricultural University, CHINA

## Abstract

The potent antimalarial drug artemisinin has a high cost, since its only viable source to date is *Artemisia annua* (0.01–0.8% DW). There is therefore an urgent need to design new strategies to increase its production or to find alternative sources. In the current study, *Artemisia carvifolia* Buch was selected with the aim of detecting artemisinin and then enhancing the production of the target compound and its derivatives. These metabolites were determined by LC-MS in the shoots of *A*. *carvifolia* wild type plants at the following concentrations: artemisinin (8μg/g), artesunate (2.24μg/g), dihydroartemisinin (13.6μg/g) and artemether (12.8μg/g). Genetic transformation of *A*. *carvifolia* was carried out with *Agrobacterium tumefaciens* GV3101 harboring the *rol* B and *rol* C genes. Artemisinin content increased 3-7-fold in transgenics bearing the *rol B* gene, and 2.3-6-fold in those with the *rol C* gene. A similar pattern was observed for artemisinin analogues. The dynamics of artemisinin content in transgenics and wild type *A*.*carvifolia* was also correlated with the expression of genes involved in its biosynthesis. Real time qPCR analysis revealed the differential expression of genes involved in artemisinin biosynthesis, i.e. those encoding amorpha-4, 11 diene synthase (ADS), cytochrome P450 (CYP71AV1), and aldehyde dehydrogenase 1 (ALDH1), with a relatively higher transcript level found in transgenics than in the wild type plant. Also, the gene related to trichome development and sesquiterpenoid biosynthesis (TFAR1) showed an altered expression in the transgenics compared to wild type *A*.*carvifolia*, which was in accordance with the trichome density of the respective plants. The trichome index was significantly higher in the *rol B* and *rol* C gene-expressing transgenics with an increased production of artemisinin, thereby demonstrating that the *rol* genes are effective inducers of plant secondary metabolism.

## Introduction

Artemisinin (AN), a sesquiterpene lactone produced mainly in *Artemisia annua* as a secondary metabolite, is a highly effective natural product against malaria and other parasitic diseases, as well as an anti-cancer agent. The World Health Organization (WHO) has recommended the Artemisinin-based Combination Therapy (ACT) for the treatment of malaria [1,2]. Our group has previously reported artemisinin in 12 *Artemisia* species found in Pakistan [3] but at very low levels, i.e. 0.01–0.8% of the dry weight of the plant [4], reaching a maximum of 1.5% in some cases [5,6]. There is therefore a need either to enhance the metabolite concentration in the natural plant or seek alternative sources of artemisinin.

Among different strategies used to improve secondary metabolite production, the recombinant DNA technology has made it possible to directly modify the expression of biosynthetic genes, and manipulate the pathways that lead to secondary plant compounds [7]. Various studies have shown *rol* genes to be powerful inducers of secondary metabolism in different plant families [8]. *Rol* A is a DNA-binding protein and stimulator of growth, while the tyrosine phosphatase activity of *rol B* regulates the signal transduction pathway of auxin [9]. *Rol B* has been used to increase the production of resveratrol in *Vitis amurensis* [10] and anthraquinones in *Rubia cardifolia* [11]. The *rol* C gene, which has cytokinin glucosidase activity, is capable of stimulating the production of many secondary compounds in transformed plants and cell cultures, such as tropane alkaloids, pyridine alkaloids, indole alkaloids, ginsenosides and anthraquinones [12,13,14]. Our previous work also showed that *Artemisia dubia*, when transformed with *rol ABC* genes, produces 10 times more artemisinin and its derivatives [15].

**Fig 1:**
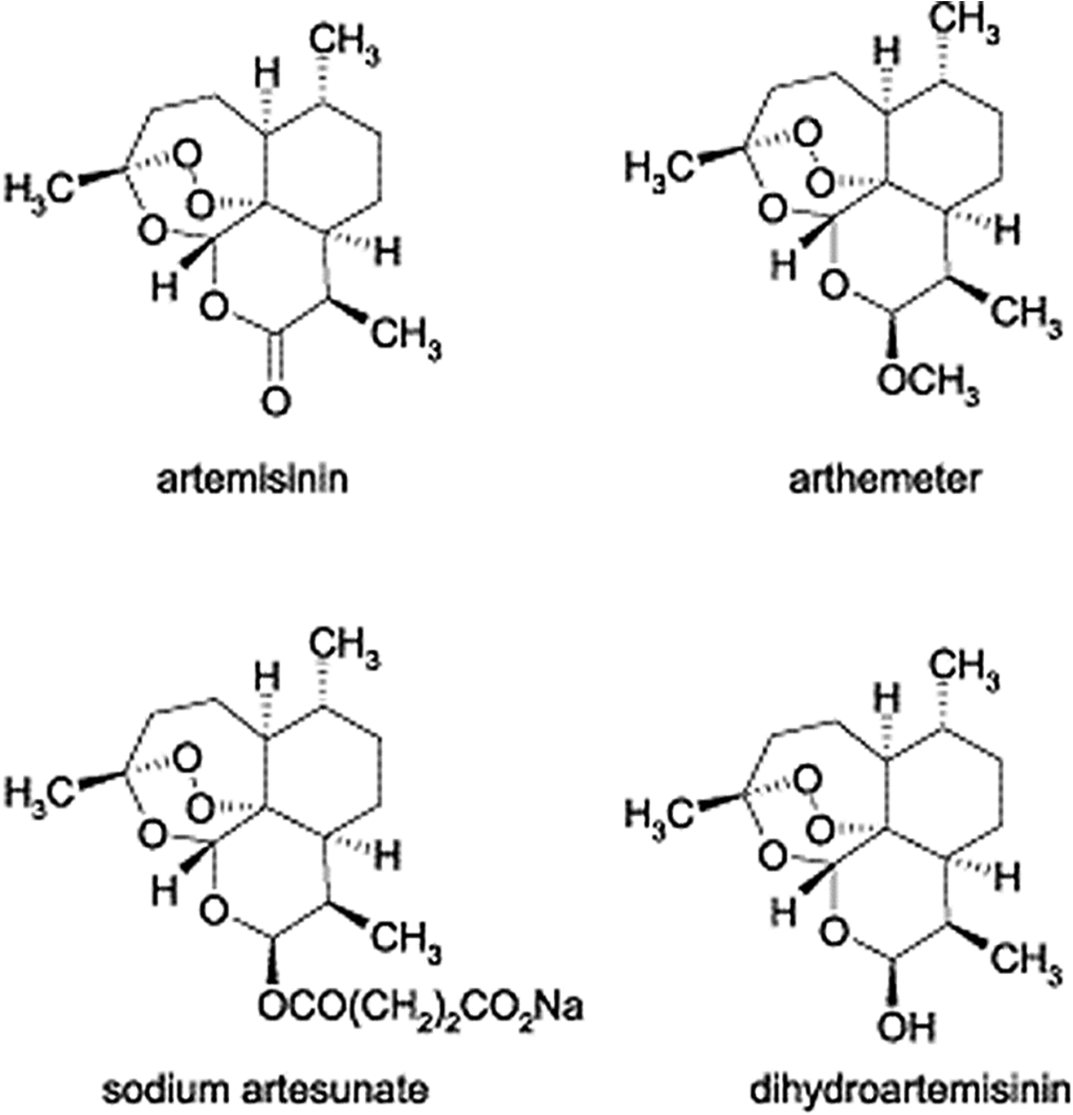
Artemisinin and derivatives. Structure of artemisinin and derivatives studied. Fig taken from literature [16].

**Fig 2:**
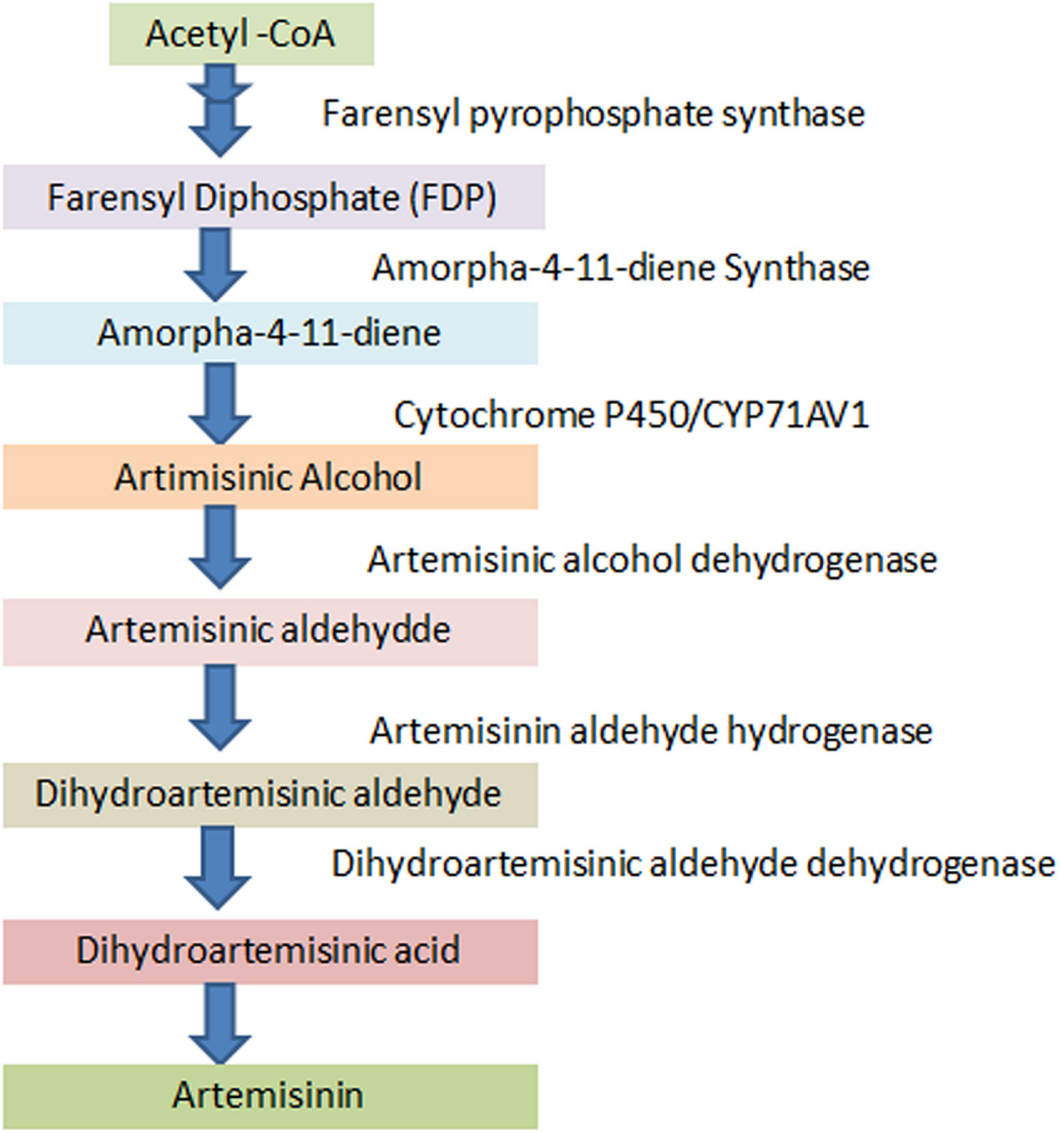
Artemisinin biosynthesis. A schematic diagram of artemisinin biosynthetic pathway.

The aim of the present investigation, after the detection of artemisinin and its derivatives [16] (Fig 1) in *Artemisia carvifolia* Buch, was to enhance the levels of these metabolites by the expression of *rol* B and *rol* C genes. As the *A*. *carvifolia* Buch transgenics showed a significant increase in target compound levels, real time qPCR analysis of artemisinin biosynthetic genes including those encoding amorpha-4, 11 diene synthase (ADS), cytochrome P450 (CYP71AV1), and aldehyde dehydrogenase 1 (ALDH1) [17,18,19,20] (Fig 2), was carried out to find a correlation between the level of gene expression and metabolite concentration. As artemisinin and other secondary metabolites are produced in 10-celled glandular trichomes located on leaves and other aerial parts [21,22,23] and sequestered in the epicuticular sac at the apex of the trichome [22], we also carried out RT-qPCR analysis of the TAFR1 (trichome-specific fatty acyl-CoA reductase 1) gene. TAFR1 is an enzyme involved in trichome development and sesquiterpenoid biosynthesis. We also calculated the trichome density to find a relationship between trichome development and artemisinin production.

## Material and Methods

### Seed Germination and DNA Barcoding

Seeds of *Artemisia carvifolia* were collected from Astore, in the Northern regions of Pakistan (35.3667° N, 74.8500° E; 8,500 ft elevation). No specific permissions were required for the mentioned location for collection of seeds. However, the project was approved by the Institutional Biosafety Committee (IBC) Quaid-i-Azam University Islamabad, Pakistan. After collection, seeds were surface sterilized with 70% ethanol and germinated on half-strength MS medium. Genomic DNA was extracted from the germinated plantlets according to our established lab protocol [24]. For the identification of *A*.*carvifolia* Buch, a non-coding spacer between the *psbA* and *trnH* genes of chloroplast DNA was amplified by PCR using primers of *psbA*: 5′ -GTTATGCATGAACGTAATGCTC-3′; *trnH*: 5'-CGCGCATGGTGGATTCACAATC-3'. The PCR reaction was carried out according to the reaction conditions reported previously [25]. Rapid PCR Purification System 9700 (Marligen Biosciences, Ijamsville, MD, USA) was used to purify the PCR product, which was then sequenced following the dideoxy-chain termination method using an ABI Prism 310 Automated DNA Sequencer (PE, Applied Biosystems, Foster City, CA, USA). Sequences were identified and aligned via the BioEdit sequence alignment tool (editor version 7.2.5.0).

### Bacterial Strains and Plasmids

**Fig 3:**
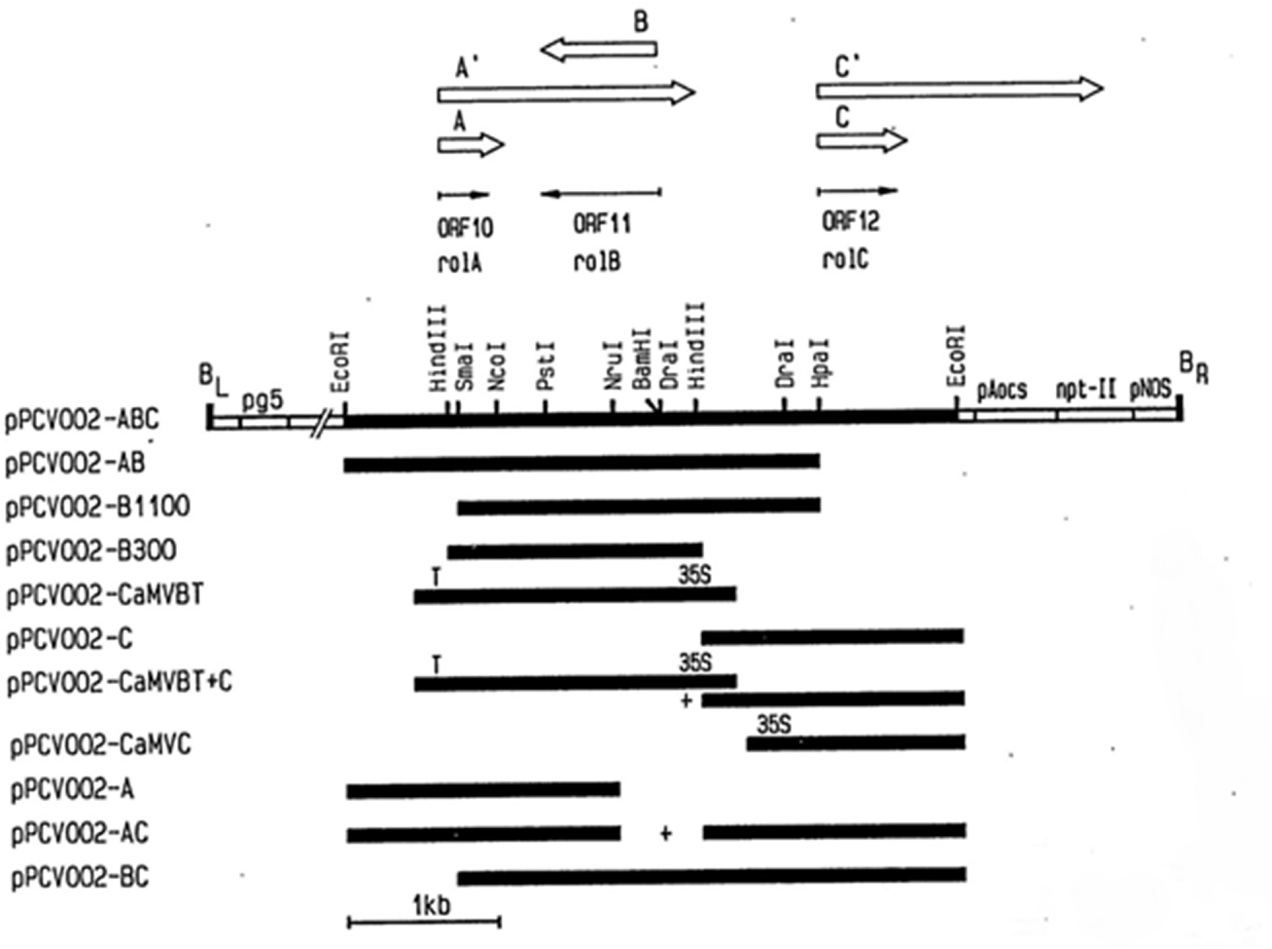
Detail of vectors used for transformation of *A*. *carvifolia*. Vectors (pPCV002-CaMVBT and pPCV002-CaMVC) used in the transformation of *Artemisia carvifolia*. Fig taken from literature [26].

*Agrobacterium tumefaciens* strain GV3101 containing plasmids pPCV002-CaMVBT and pPCV002-CaMVC, kindly provided by Dr. A. Spena, Max-Planck-Institut fur Zuchtungsforschung, 5000 Koin 30, FRG [26], was used for transformation purposes. The coding sequence of the *rol* B and rol C genes was expressed in the T-DNA region of the plasmids pPCV002-CaMVBT and pPCV002-CaMVC, respectively, under the control of the CaM35S promoter. T-DNA of pPCV002-CaMVBT and pPCV002-CaMVC also contained the *neomycin phosphotransferase* (*NPTII*) gene with the nopaline synthase (NOS) promoter and NOS terminator sequences (Fig 3). *Agrobacterium tumefaciens* containing the plasmids pPCV002-CaMVBT and pPCV002-CaMVC were grown overnight in Luria broth (Sigma Cat # L-1900). After inoculation, bacterial cultures were maintained at 28°C and 120 rpm in a shaking incubator. Growth was obtained in 24 hours and OD was checked by spectrophotometer, when in the range of 0.2–0.8, the bacterial suspension was used for the transformation.

### Transformation and Regeneration

One-month-old *in vitro* grown plants were used for transformation. Nodal, explants were prepared, and after 2 days of preculturing on shooting media (0.5 mg/l BAP and 0.1 mg/l NAA) supplemented with 200μM acetosyringone, they were infected with bacterial strains containing the desired constructs. After 10–15 minutes of bacterial infection, the explants were dried on autoclaved filter paper for 2–3 minutes and placed on MS shooting media supplemented with 200μM acetosyringone. After 2 days of incubation in darkness at 28°C, explants were washed with antibiotics and placed on selection medium (0.5mg/l BAP, 0.1mg/l NAA, 50mg/l kanamycin, 300mg/l cefotaxime). Regeneration occurred within one month, and explants were shifted to fresh selection media every 15 days. When shoots attained considerable length of 3-4cm, they were shifted to rooting media (NAA 0.1mg/l, kanamycin 50mg/l) to get complete plant with roots. After three to four selection cycles, the complete plants were regenerated on selection media.

### Molecular Analysis

Molecular analysis was performed after extraction of genomic DNA from aerial parts of 2 month old transformed and wild type plants by the CTAB method [24]. The plasmid from GV3101 was isolated by the alkaline lysis method. PCR analysis was performed using a programmed DNA thermal cycler (Biometra, USA). The *rol* B gene forward 5’-GCTCTTGCAGTGCTAGATTT-3’ and reverse primer 5’-GAAGGTGCAAGCTACCTCTC-3’, the *rol* C gene forward 5’-GAAGACGACCTGTGTTCTC-3’ and reverse primer 5’- CGTTCAAACGTTAGCCGATT-3’ and the *nptII* gene forward 5’-AAGATGGATTGCACGCAGGTC-3’ and reverse primer 5´GAAGAACTCGTCAAGAAGGCG-3´ were used for PCR analysis. Conditions applied for PCR were as described previously [15].

### Southern Blotting

Southern blot analysis was performed using the DIG High Prime DNA Labeling and Detection Starter Kit II (Roche Cat. No. 11585614910), following the manufacturer’s instructions. For southern blotting, 1–5 μg of genomic DNA of *rol B* transformants was digested with X-baI and that of *rol C* gene transformants was digested with EcoRI and electrophoresed on 0.7% agarose gel overnight. The transfer of digested DNA to the positively charged nylon membranes was carried out according to the standard procedure [27]. The probe used for hybridization was the PCR products of *rol B* and *rol C* genes from plasmids, which was labeled using digoxigenin (DIG)-11-dUTP with DIG High Prime DNA Labeling reagents (Roche, Mannheim, Germany). Hybridization was done at 42–44°C and then immunological detection was carried out on X-ray film using CSPD substrate in accordance with the manufacturer’s instructions.

### Analysis of Artemisinin and Its Derivatives by HPLC-ESI/MS

Extraction of artemisinin and its derivatives from shoots of 4 months old transformed and wild type plants was carried out using the reported methodology [28]. HPLC-MS analysis was performed with a Varian 500 Ion Trap Mass spectrometer (Varian, Spain) coupled to a 212-HPLC system equipped with an auto sampler 410 (Varian, Spain). The analytes were separated by a C18 (5μm) column (150mmx 4.6mm) (AKDAY Chromatografica, Spain) using a mobile phase consisting of water with 0.1% formic acid (A) and acetonitrile (B) in a gradient as follows: (t (min), % B) (0, 50) (10, 50) (15, 99) (23, 50) (26, 50). The flow rate was 1ml/min. Electro spray ionization (ESI) in positive mode was applied with a capillary voltage of 4000V and plate voltage of 600V. Other parameters include: spray chamber temperature 50 C, nebulizing gas (N_2_) pressure: 35psi, dry gas (N_2_) 10 l/min at 35psi and 400C. Mass analyzer scanned from 66–500 u. Fragmentation amplitude was 1.0 V. Three injections per sample were applied.

### Expression Analysis of *rol* Genes and Artemisinin Biosynthetic Genes by Real Time Quantitative PCR

The young leaves of transformed and wild-type plants (4 months old) were selected for the RT-qPCR analysis. Total RNA was extracted from three replicates of each plant according to the procedure reported previously [29] and also by using the trizol reagent (Life Technologies). Turbo DNAse (Ambion) was used to ensure complete removal of DNA from RNA after extraction. Purity and quantity were checked by taking absorbance at 260 and 280nm on a Nanodrop ND-2000 spectrophotometer (Thermo scientific). The quality of RNA was also assessed by running RNA samples on 1.2% agarose gel. Reverse transcription of 1 μg of RNA was carried out using a 1^st^ strand cDNA synthesis kit (Invitrogen), following the manufacturer’s instructions. In order to check the expression of *rol* B and *rol* C genes, a semi-quantitative reverse transcriptase-polymerase chain reaction was performed with *rol* B and *rol* C gene primers as previously, using 1 μl of cDNA reaction mixture as a template. The PCR reactions were run in triplicate. Gel images of PCR products were scanned by Kodak Molecular Imaging software (version 4.2) and integrated density values were found to be different for each band.

To evaluate the possible effects of *rol* genes on the expression of artemisinin biosynthetic genes, quantitative real time PCR of four selected genes was performed, namely those encoding amorpha-4, 11 diene synthase (ADS), cytochrome P450 (CYP71AV1) and aldehyde dehydrogenase 1 (ALDH1). The gene involved in trichome development and sesquiterpenoid biosynthesis (TFAR1) was also studied. The β-actin gene was used as reference gene [30]. For real time qPCR, a 1:4 dilution of cDNA was used. The amplification reaction was performed by gene specific primers i.e. ADS forward: 5’-ATTACTGGCGGTGCTAAC-3’ and reverse: 5’-GTGCAGAGACAGCCCATT-3’, CYP71AV1 forward: 5’-ATTTTGGATATGTTTGGAGCAGGC-3’ and reverse: 5’-TCCGCTTGTACTTTCTCCATTGCT-3’, ALDH1 forward: 5’-CAGGAGCTAATGGAAGTTCTAAGTCAG-3’ and reverse: 5’-TTTCTTCCTTCGGCCACTGTTG-3’, TAFR1 forward: 5’-CCTTGGAGATCCTGAAGCTG-3’ and reverse: 5’-CGTTGGATTGTGCTGAACTG-3’, β-actin forward: 5’-ATCAGCAATACCAGGGAACATAGT-3’ and reverse: 5’-AGGTGCCCTGAGGTCTTGTTCC-3’. Primer amplification efficiency was determined as previously [31]. The qPCR was performed using iTAqTM Universal SYBR Green Supermix (BioRad, Hercules, CA, USA) in a 384-well platform system (ABI Prism® 7900HT sequence detection system, Applied Biosystems, Foster, CA, USA). The reaction conditions for real time qPCR were as follows: denaturation for 5 min at 95 ◦C, followed by 45 cycles each of denaturation for 10s at 95 ◦C, annealing for 10 s at 60 ◦C, followed by elongation for 10 s at 72 ◦C. The RT-qPCR reactions were run in triplicate with mean values and standard deviation calculated for all cycle thresholds (Ct). For each gene, the relative expression levels were normalized with respect to the wild-type plant (reference value = 1).

### Calculations of Trichome Density

Young leaves of wild-type and transformed *A*. *carvifolia* plants were selected to calculate trichome density. The ventral surfaces of the leaves were attached to the microscopic slides with the help of glue and maintained overnight at 4°C for dehydration. The next day, slides were examined under the fluorescent microscope (Leica) with a FITC green filter. Slides were studied under 10 x resolution and photographed.

### Statistical Analysis

All the experiments including, PCR, LC-MS and trichomes analysis were run in triplicate. Results of metabolites content, real-time quantitative PCR analysis and calculation of trichome density are presented as mean values ± S.E. The data obtained for the qualitative and quantitative analysis of artemisinin and derivatives, was analyzed statistically by ANOVA and Duncan’s multiple range test using Mstat C software. Statistical significance of trichomes was determined by t-test (**, P<0.01; *, P<0.05).

## Results

### Plant Identification through DNA Barcoding

**Fig 4:**
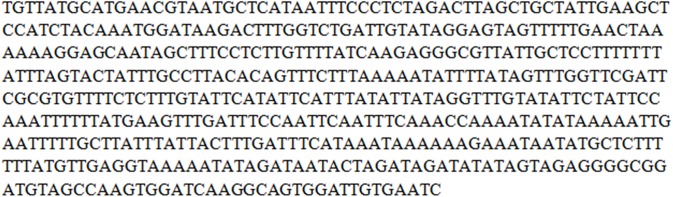
Sequenced DNA for identification of *Artemisia carvifolia*. *psbA-trnH* sequence of *A*. *carvifolia* [NCBI: FJ418751].

We successfully amplified the 500bp *psbA-trnH* region of the chloroplast genome (Fig 4). DNA samples were sequenced in triplicate to confirm the authenticity of species-specific nucleotides and the same results were obtained. GenBank Accession number [NCBI: FJ418751] was used as the reference sequence to confirm our plant species. After performing the CLUSTAL W in BioEdit software (version 7.2.5.0) and BLAST in NCBI, our sequence was confirmed as *psbA-trnH* of *A*. *carvifolia*.

### Transformation and Regeneration

**Fig 5:**
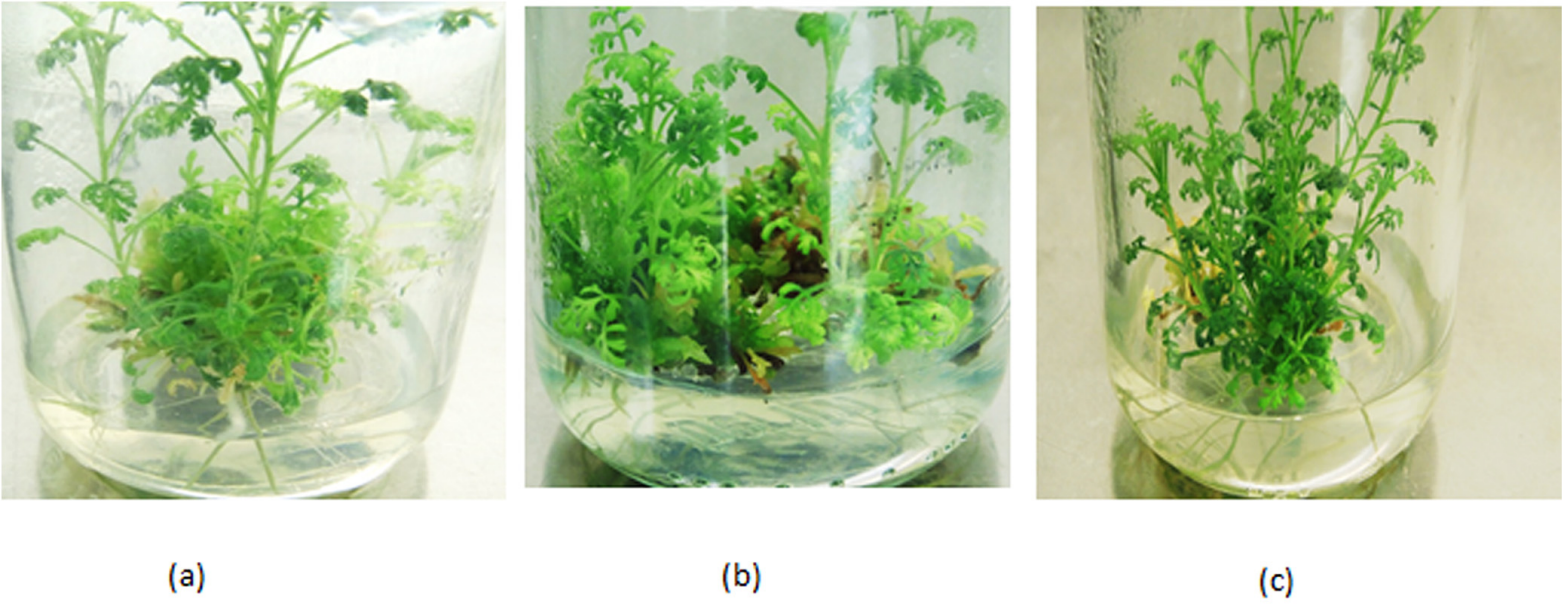
Vegetative propagation of *A*. *carvifolia*. *A*.*carvifolia*: wild type (a), tranformant of *rol* B (b), and transformant of *rol* C gene (c).

Successful transformation of *A*. *carvifolia* with *A*. *tumefaciens* GV3101 harboring the *rol B* and *rol C* genes was carried out. Two independent transformation experiments were performed and 300 explants were used per transformation event. Transformation efficiency was found to be 20–30%, but only 4 *rol B* transformants and 3 *rol C* transformants survived to maturity on the selection media. Morphological variability was observed between wild type plants and transgenics bearing *rol B* and *rol C* genes. *Rol B* transgenics, which grew faster on the selection media, had wider leaves and more inflorescence, while *rol C* transgenics, which were recalcitrant to regeneration, showed a narrow leaf blade and decreased internode length (Fig 5).

### Molecular Analysis

**Fig 6:**
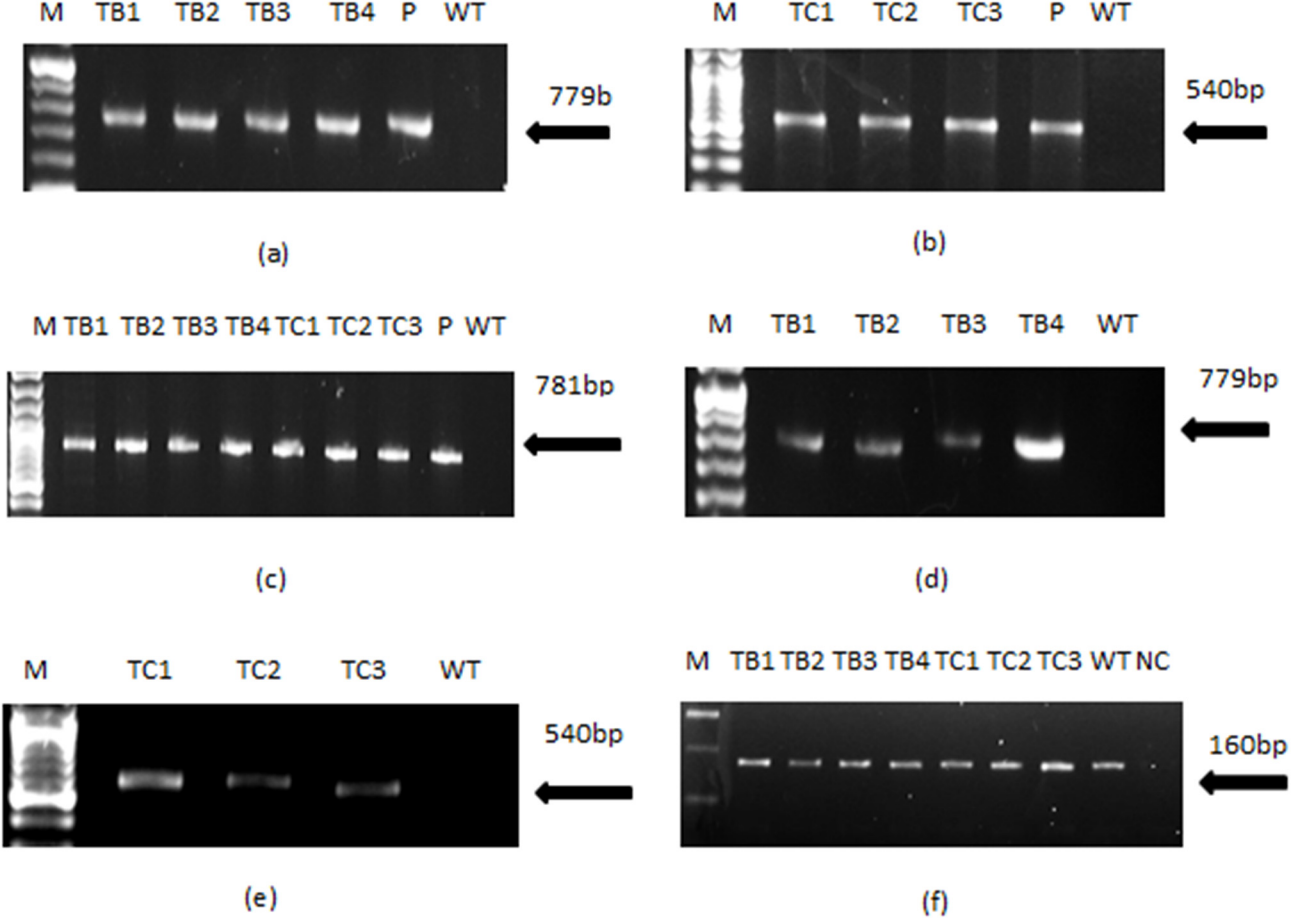
Gel pictures showing results of normal and semi quantitative reverse transcriptase PCR. PCR amplified products of *rol* B (a), *rol* C (b) and *nptII* (c) gene are shown in the figure. Semi quantitative RT-PCR showing the relative expression of *rol B* (d) and *rol C* gene (e). β-actin used as housekeeping gene (f). TB1-TB4 represents *rol* b transgenics whereas TC1-TC3 represent *rol* C transgenics. WT stands for wild type plant of *A*. *carvifolia*, lane “P” refers to the plasmid DNA and lane M corresponds to the marker DNA (Fermentas). NC = negative control.

**Fig 7:**
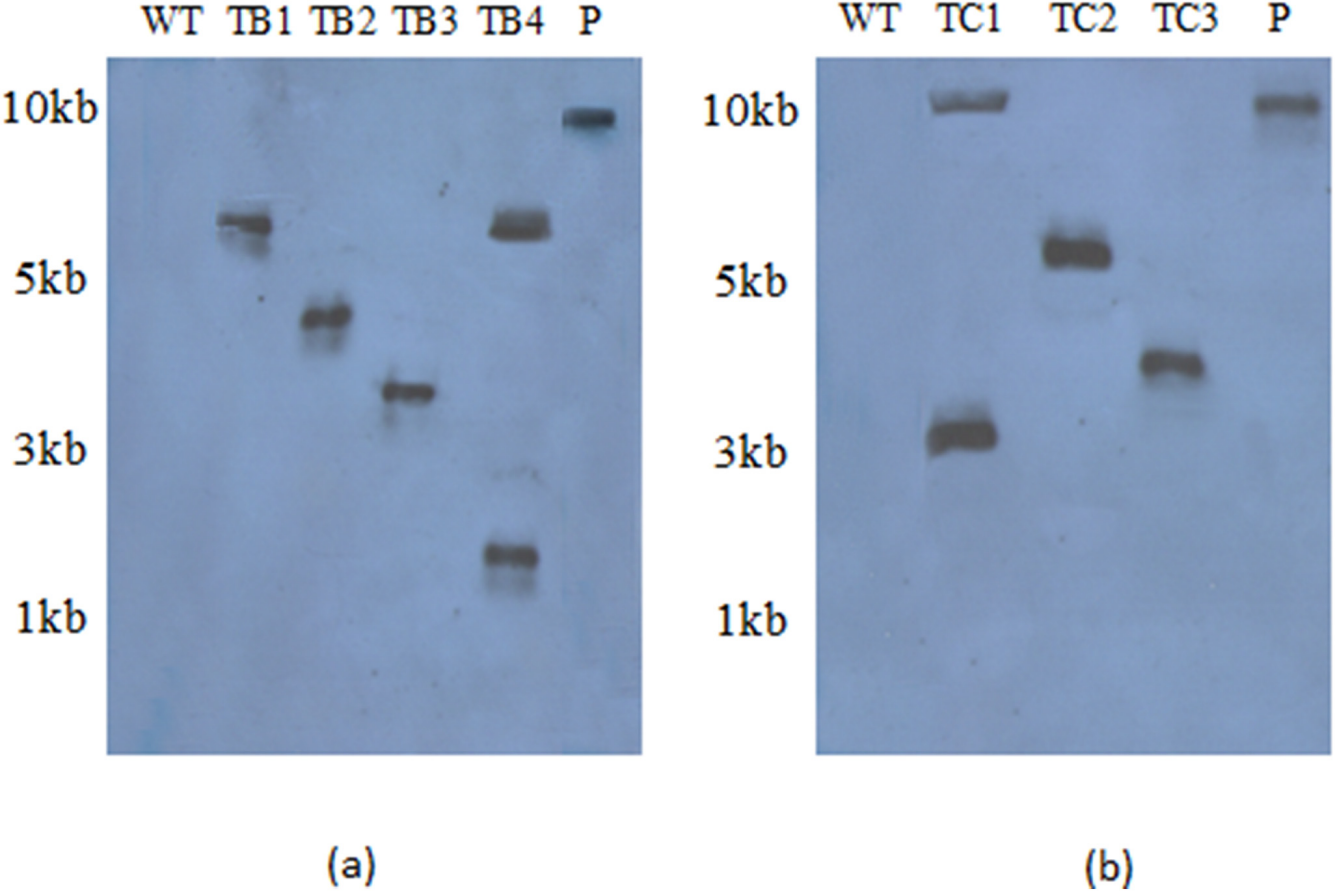
Southern blot analysis. Southern blot analysis of *rol* B (a) and *rol* C transgenic lines (b). TB1-TB4 represents *rol* B transgenics whereas TC1-TC3 represent *rol* C transgenics. Whereas "P” stands for the plasmid DNA run as positive control. WT indicates the wild type plant.

PCR performed for the *rol* gene transformants of *A*.*carvifolia* showed the amplified products of 779bp for *rol B*, 540bp for *rol C* and 781bp for the *nptII* gene, as shown in Fig 6A, 6B & 6C. Similar amplified products were obtained from plasmid DNA of GV3101-CaMVBT and GV3101-CaMVC, respectively. Wild-type plants did not show the presence of these genes in their genome. Southern blot analysis confirmed the integration of *rol* genes in the plant genome and also gave an idea of the gene copy number in independent transgenic lines (Fig 7A & 7B). RT-PCR confirmed gene expression in all the regenerants, although it varied according to line. The *rol B* transgenic line TB4 and the *rol C* transgenic line TC1 showed the highest levels of expression (Fig 6D & 6E), and were found to harbor two copies of the respective genes.

### Analysis of Artemisinin and Derivatives by LC-MS

**Fig 8:**
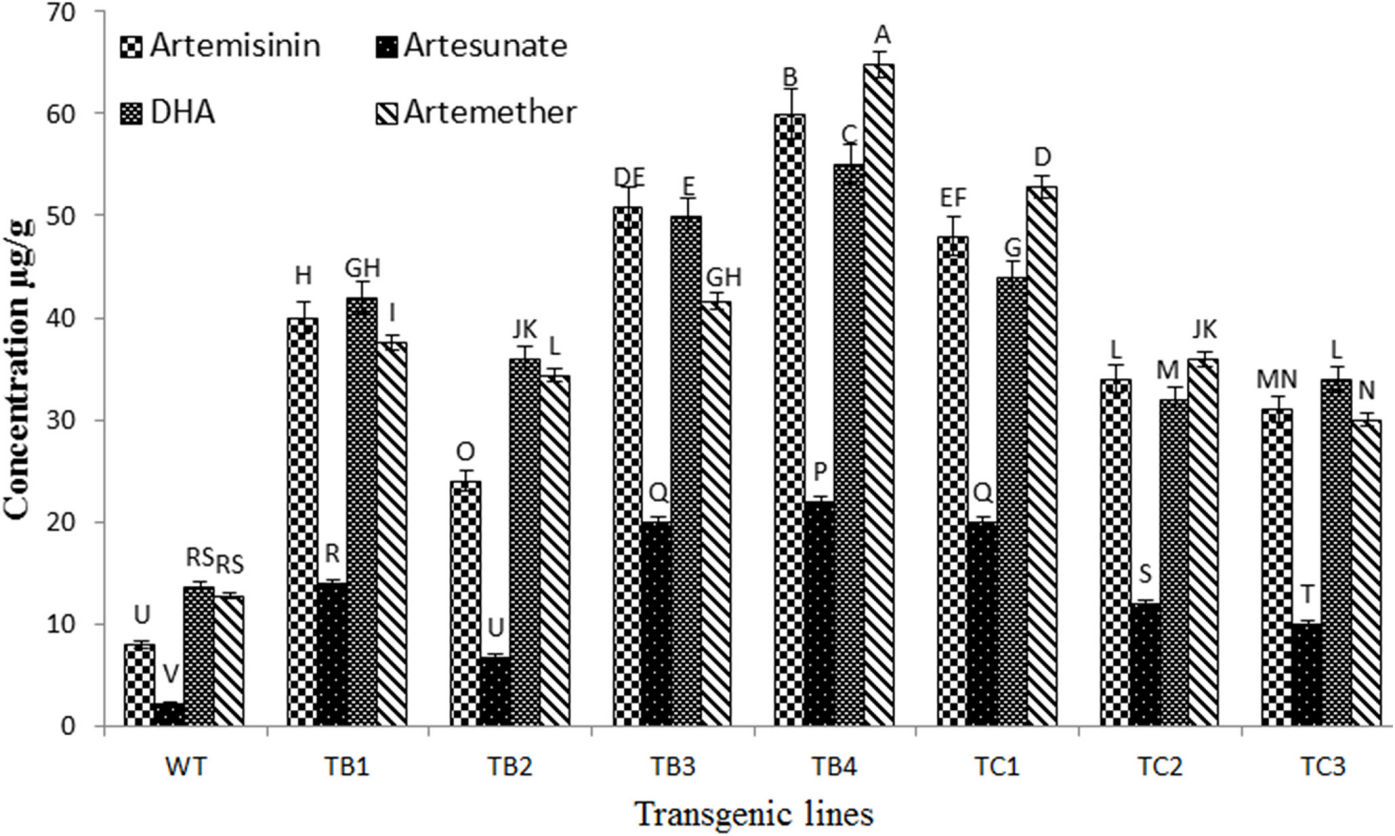
Quantitative analysis of artemisinin and derivatives. Comparative analysis of artemisinin content and its derivatives in wild type *A*. *carvifolia* and transgenics of *rol* B and *rol* C gene. Values with same alphabet did not differ significantly at 5% probability level using LSD. Error bars indicate the standard deviation.

Analysis of artemisinin and its derivatives by an HPLC-UV system was not successful in *A*. *carvifolia* plant extracts, as these compounds lack UV properties or fluorescent chromophores. Thus, a method coupling HPLC with an Ion trap mass spectrometer was developed for the detection and quantification of the target compounds (S1 Fig), with the following results found in *A*.*carvifolia* wild-type extracts: artemisinin (8μg/g), artesunate (2.24μg/g), dihydroartemisinin (D.H.A. 13.6μg/g) and artemether (12.8μg/g). Differences in content between transformed and untransformed plants were observed. All the transgenic lines bearing *rol B* and *rol C* genes showed enhanced levels of artemisinin and its derivatives (Fig 8). In *rol B* gene transgenics, this increase was 3-7-fold for artemisinin, 3–10 fold for artesunate, and 2.6–4 fold for D.H.A and artemether, whereas in *rol C* transgenics it was 3.8–6 fold for artemisinin, 4.4–8.9 fold for artesunate, 2.3–3.2 fold for D.H.A and 2.3–5 fold for artemether.

Statistical analysis was conducted in factorial design (8 X 4 X 3) to see the effect of different transgenic lines on the production of artemisinin and derivatives, where wild type *A*. *carvifolia* and transgenics of *rol B* and *rol C* gene showed significant difference (P<0.05) in the production of studied compounds (Table 1).

**Table 1:** Analysis of variance for quantitative analysis of artemisinin and derivatives.

Source	Degree of freedom	Sum of Squares	Mean Square	F-Value	Probability
Factor A (Transgenic lines)	7	12415.863	1773.695	1457.1584	0.0000
Factor B (artemisinin standards)	3	11897.913	3965.971	3258.1977	0.0000
A X B (Transgenic lines X artemisinin standards)	21	3329.692	158.557	130.2605	0.0000
Error	64	77.903	1.217		
Total	95	27721.371			

Coefficient of Variation: 3.38%

### Expression Analysis of Artemisinin and Trichome Biosynthetic Genes by Real Time qPCR

**Fig 9:**
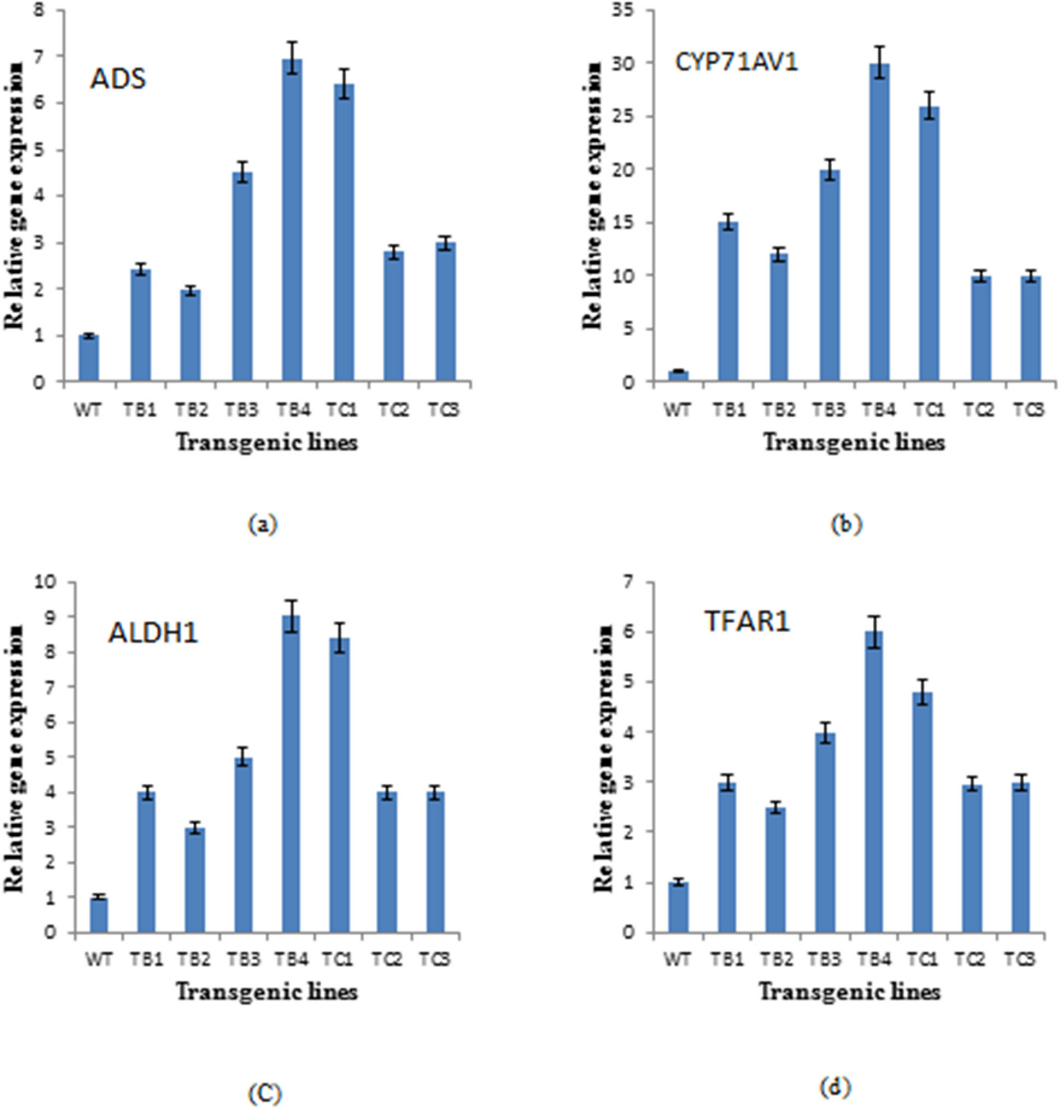
Expression analysis of artemisinin biosynthetic pathway genes and TAFR1 gene. Expression level of Artemisinin biosynthetic genes, ADS (a), CYP71AV1 (b) and ALDH1 (c) in wild type plant of *A*. *carvifolia* and transgenics of *rol B* and *rol C* gene. TFAR1 is the gene involved in trichome development and sesquiterpenoid biosynthesis (d).

Higher transcript levels of all the studied genes were found in the transgenics expressing the *rol B* and *rol C* genes, although in a variable fashion (Fig 9). Among the artemisinin biosynthetic genes, CYP71AV1 was the most expressed in the transgenics, increasing 15-30-fold in *rol B* transformants and 10-26-fold in *rol C* transformants, compared to wild-type plants. The ALDH1 and ADS genes showed a respective 3-9-fold and 2-6-fold enhanced expression in *rol B* transformants, and a 5-9-fold and 3-6-fold increase in *rol C* gene transformants. The transgenic lines TB4 and TC1 exhibited the highest expression of these genes. Real time qPCR analysis of the TFAR1 gene revealed variable expression among the transgenics, with a 3-6-fold and 2-4-fold increase, respectively, in *rol B* and *rol C* transformants.

### The Trichome Density

**Fig 10:**
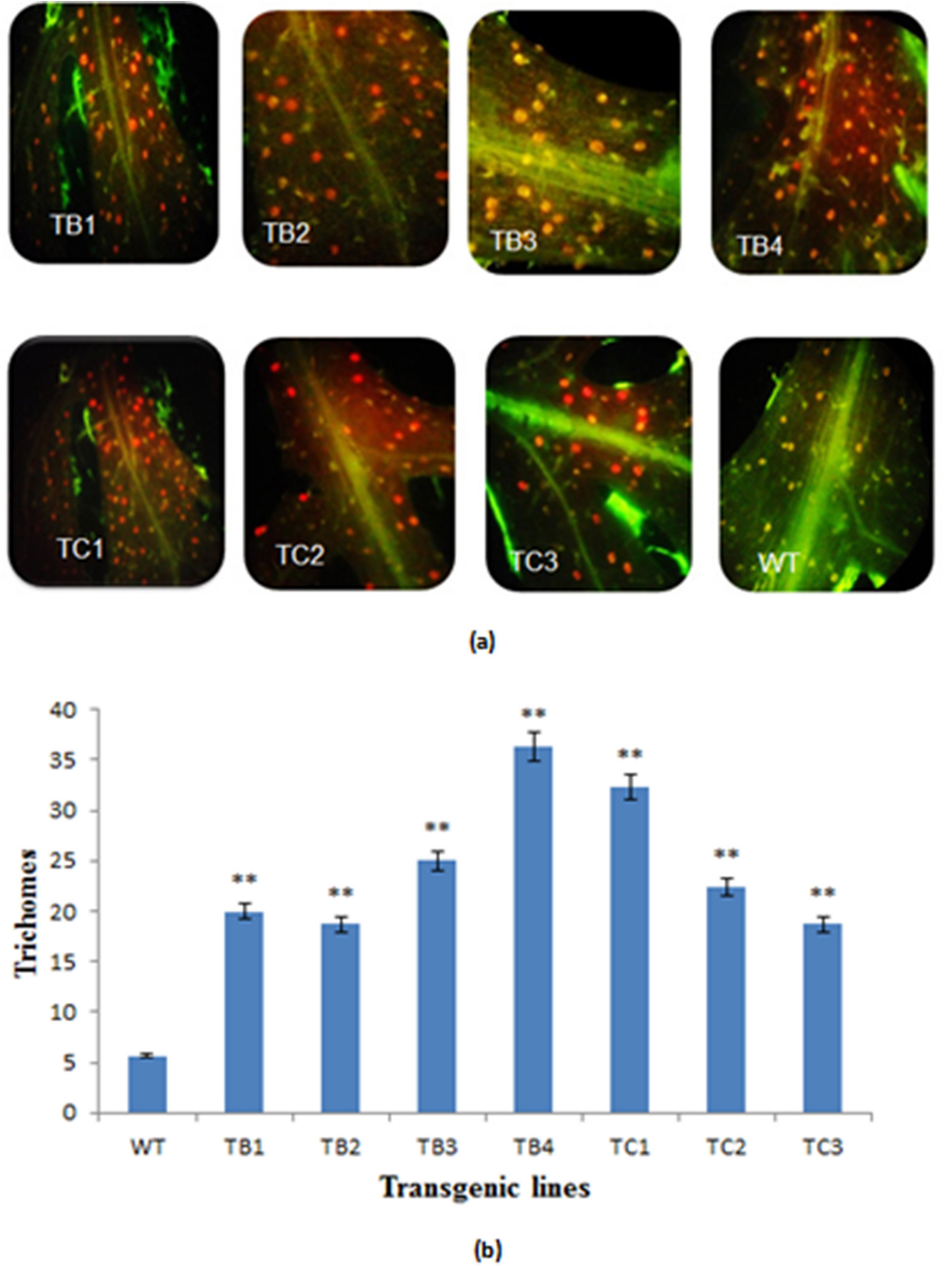
Trichome density. Comparison of trichome density of transformed and untransformed plants of *A*. *carvifolia* (a). Graphical representation of trichomes densitiy of wild type *A*. *carvifolia* and transformants of *rol B* and *rol C* gene (b). TB1-TB4 represent *rol B* transgenics whereas TC1-TC3 represent transgenics of *rol C* gene. WT represents the control plant of *A*. *carvifolia*.

The number of glandular trichomes was determined on the adaxial side of 3 random pieces of fresh leaf material from each sample, with an accurately determined leaf area of approximately 1cm^2^ per leaf, and significant differences were observed, as shown in Fig 10A & 10B. The leaves of *A*. *carvifolia* Buch transformed with *rol B* and *rol C* genes produced more trichomes (20–30 trichomes/cm^2^) compared to untransformed leaves (5–7 trichomes/ cm^2^).

## Discussion

In the *Artemisia* genus, many species have been evaluated for their artemisinin content, as mentioned previously, but not *A*. *carvifolia*. We not only detected this important phytochemical and its derivatives in this species, but also demonstrated the effectiveness of *rol* genes in the enhancement of their production.

Transformants harboring the *rol B* and *rol C* genes showed a significant increase in their artemisinin content compared to the wild-type plants. Data was obtained from four independent regenerants bearing *rol B* and three independent regenerants bearing *rol C* genes from two independent transformation events. Significant differences were observed in terms of growth, morphology and artemisinin accumulation. Similar findings were reported by another group who showed for the first time how *rol* genes induce ginsenoside overproduction in transformed cell cultures of *P*. *ginseng*, using plasmid DNA containing the individual *rol* genes from the TL-DNA of *A*. *rhizogenes* strain A4 [32]. *Rol C* cultures were found to accumulate 1.8-3-fold more ginsenoside than the control plant, although *rol B* lines were less productive [32]. In contrast, in another study, *rol B* gene transgenics of *Rubia cardifolia* showed enhanced production of anthraquinones. In fact, β-glucosidase, the product of the *rol B* gene, releases indole acetic acid (IAA) from its inactive glucose conjugates, thus increasing auxin sensitivity in *rol B* transgenics. Increased tyrosine phosphatase activity in *rol B* transformed cells distracts the signal transduction pathway of the hormone. This sensitivity and transduction alters the physiological configuration of transformed cells and eventually the whole plant [11,33,34,35,36,37,38]. Results of current study are also supported by our previous report, demonstrating the effect of *rol ABC* gene construct in over production of artemisinin in *A*. *dubia* [15]. It may be concluded that *rol* genes when combined show more increase in artemisinin i.e. up to 10 fold [15] than individual *rol* genes as we observed 6–7 fold increase.

Morphological alterations and differences in metabolite concentrations among different transgenic lines have been related to the variability in the expression level of *rol* genes and can be attributed to position effect [39]. Transgenic *A*. *carvifolia* plants showing increased secondary metabolism and morphological alterations were analyzed by Southern blot analysis and semi quantitative RT-PCR to check for correlation with *rol* gene expression. The results confirmed that the observed changes were due to the presence of *rol* gene transcripts. The lines TB4 and TC1, which produced the most artemisinin, also showed more *rol* B and *rol* C gene transcripts, respectively, as well as bearing two copies of these genes. On the other hand, the unimproved accumulation of artemisinin and derivatives observed in TB2 can be attributed to the less *rol B* transcripts in this line. Identical conclusions were reached by other group who correlated the altered morphology of tobacco plants with the detectable level of *rol* gene transcripts [39]. Our findings are also supported by Arshad et al. (2014), who found that the expression of the *rol* B gene of *A*. *rhizogenes* in tomato enhanced nutritional contents and foliar tolerance against fungal pathogens [40].

Variation in the target compound accumulation also reflected differential expression of artemisinin biosynthetic genes, with transcript levels being higher in the *rol B* and *rol C* gene transformants than in wild-type *A*. *carvifolia*. Key enzymes in artemisinin biosynthesis include ADS, CYP71AV1 and ALDH1, which are involved in the conversion of farensyl diphosphate (FDP) to artemisininc acid, a late precursor of artemisinin. Biochemical and molecular studies have shown that amorpha-4,11-diene synthase (ADS) catalyzes the first committed step of the artemisinin biosynthetic pathway by cyclization of FDP to amorpha-4,11-diene [41,42,43,44,45], which is then oxidized to artemisinic alcohol by the cytochrome P450 enzyme, CYP71AV [18,46]. Conversion of artemisinic alcohol to artemisinin through formation of either artemisinic aldehyde or dihydroartemisinic aldehyde is catalyzed by the enzyme ALDH1. *A*. *carvifolia* transgenics bearing *rol B* and *rol C* genes showed an overexpression of the studied genes, particularly CYP71AV, in accordance with the accumulation of artemisinin and its derivatives. These results are supported by a previous study showing that overexpression of farensyl pyrophosphate synthase (FPS) in *A*. *annua* increases the accumulation of artemisinin through conversion of isopentenyl diphosphate (IPP) and dimethylallyl diphosphate (DMADP) into FDP [47]. Similarly, an increased amount of artemisinin was reported in *A*. *annua* transformed with *Agrobacterium tumefaciens* wild type nopaline strains [48], and in *A*. *annua* bearing the *Ipt* gene [49].

The TFAR1 gene, which stimulates trichome development and catalyzes sesquiterpenoid biosynthesis [47], also showed increased expression in all transgenic lines. Accordingly, the transformed lines of *A*. *carvifolia* showed higher trichome density compared to the untransformed plant (Fig 10), indicating a relationship between trichome development and artemisinin production. Glandular trichomes are the sites of production of important phytochemicals, including artemisinin, as well as many others that have found numerous applications in the pesticide, pharmaceutical and flavor industries [50,51]. Artemisinin content has been directly related to the trichome index in previous studies [52,53].

## Conclusion

Transformation of *A*. *carvifolia* with *rol B* and *rol C* genes resulted in the enhancement of its secondary metabolites, particularly artemisinin and its derivatives. Transcript levels of the *rol B* and *rol C* genes found in the transgenics also correlated with their artemisinin accumulation pattern. An altered expression of genes involved in artemisinin and trichome biosynthesis was observed, indicating that *rol* gene integration modified the plant metabolism. The relationship between trichome density and accumulation of artemisinin and its derivatives was confirmed.

## Supporting Information

S1 Fig LC-MS chromatograms. LC-MS chromatograms for standard artemisinin, artesunate, D.H.A and artemether for C18, 5μm (150mm x 4.6mm) column along with their mass spectra.
(TIF)
