# Supplementary figures and images for: Genetic Transformation of Artemisia carvifolia Buch with rol Genes Enhances Artemisinin Accumulation
Source: PLoS One. 2015 Oct 7;10(10):e0140266. doi: 10.1371/journal.pone.0140266 (PMC4596866; doi:10.1371/journal.pone.0140266)

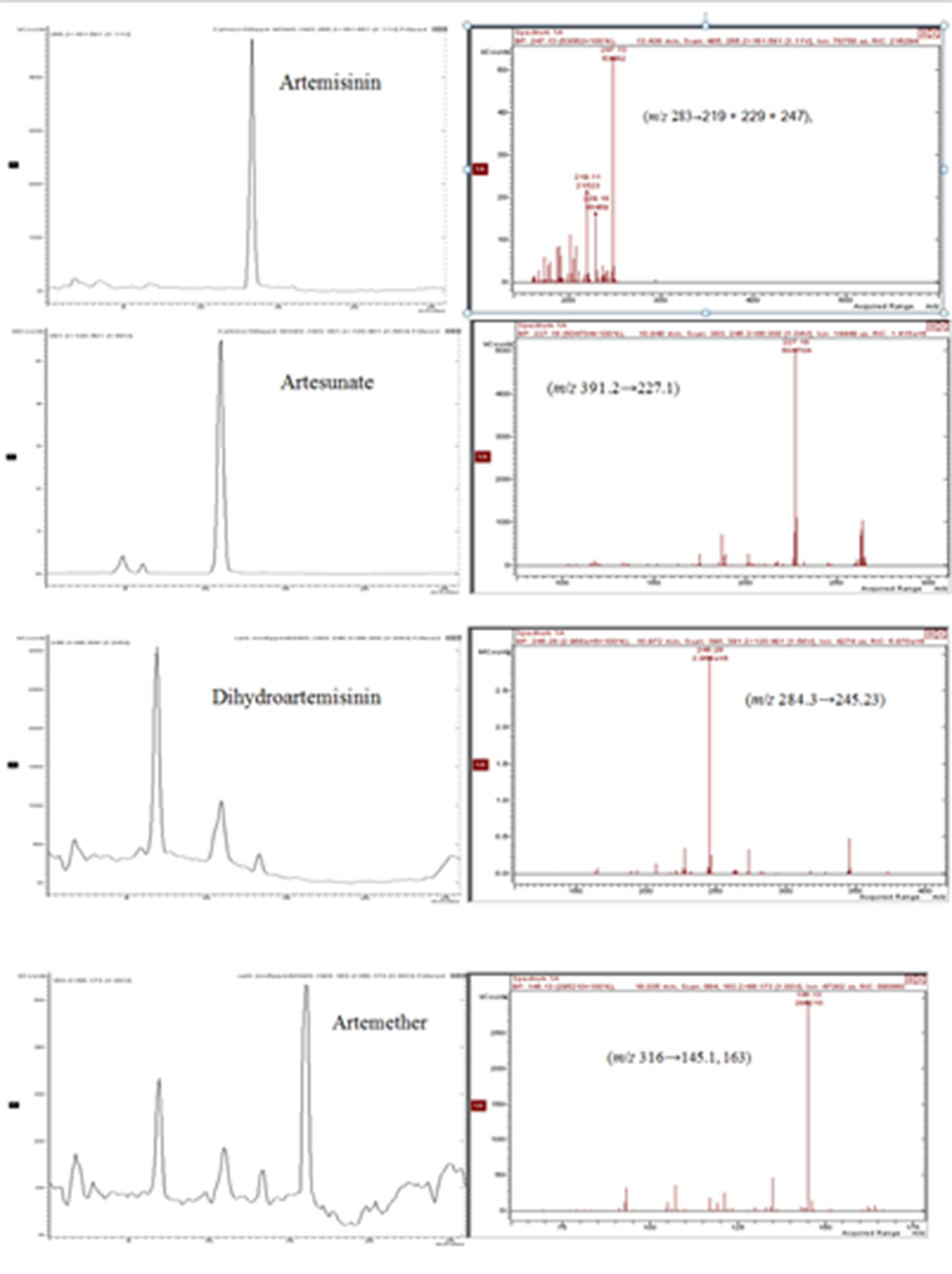

Supplement: S1 Fig — LC-MS chromatograms for standard artemisinin, artesunate, D.H.A and artemether for C18, 5μm (150mm x 4.6mm) column along with their mass spectra. (TIF) [file pone.0140266.s001.tif]
